# Flow 2.0: A Standardized Toolbox for Real‐Time Phase‐Contrast MRI of Neurofluid Dynamics

**DOI:** 10.1002/mrm.70519

**Published:** 2026-07-22

**Authors:** Pan Liu, Olivier Balédent

**Affiliations:** ^1^ Medical Image Processing Department CHU Amiens‐Picardie University Hospital Amiens France; ^2^ CHIMERE (INSERM UA‐21), Jules Verne University of Picardie Amiens France

**Keywords:** cardiorespiratory coupling, neurofluid dynamics, phase‐resolved analysis, real‐time phase‐contrast MRI, RT‐PC

## Abstract

**Purpose:**

Real‐time phase‐contrast MRI (RT‐PC) enables continuous quantification of physiological flow dynamics across multiple temporal frequencies, but its broader application is limited by complex and fragmented post‐processing workflows. We developed Flow 2.0, a standardized toolbox for RT‐PC designed to integrate segmentation, flow quantification, visualization, and multi‐frequency signal analysis within a unified environment.

**Theory and Methods:**

Flow 2.0 integrates automated ROI segmentation, background phase correction, velocity aliasing correction, interactive waveform visualization, cardiac cycle reconstruction, and Hermitian‐preserving frequency‐domain processing. A phase‐resolved respiratory modulation module enables systematic evaluation of respiratory influences through temporal phase shifting and cycle‐based multi‐parameter analysis. The framework was demonstrated using representative RT‐PC datasets acquired under free and sustained deep breathing conditions.

**Results:**

Automated processing of 500‐frame RT‐PC datasets was completed within seconds while preserving signal phase integrity. Cardiac cycle reconstruction and respiratory phase classification were consistently applied across acquisition conditions. The phase‐resolved analysis module enabled visualization and quantification of respiratory modulation across multiple flow‐derived parameters, including mean flow, stroke volume, and cardiac‐related metrics.

**Conclusion:**

Flow 2.0 provides a standardized and extensible toolbox for RT‐PC MRI, enabling reproducible, phase‐resolved, and multi‐parametric analysis of neurofluid dynamics within a unified processing environment.

## 1 Introduction

Neurofluid dynamics studies have demonstrated coupled interactions between cerebrospinal fluid (CSF) and cerebral blood flow within the craniospinal system [1, 2, 3]. Real‐time characterization of these interactions has become an important topic in neurofluid imaging research [4, 5, 6].

Conventional cardiac‐gated phase‐contrast (CINE‐PC) MRI primarily characterizes cardiac‐frequency flow dynamics [7, 8, 9]. Real‐time phase‐contrast MRI (RT‐PC) enables continuous velocity mapping with temporal resolutions shorter than 100 ms over extended acquisition periods, allowing characterization of multi‐frequency flow dynamics, including respiratory and lower‐frequency components [2, 10, 11, 12, 13, 14], within a single acquisition without physiological gating [15, 16, 17].

Despite these advantages, RT‐PC post‐processing remains methodologically complex. RT‐PC datasets typically contain thousands of temporal frames with relatively low signal‐to‐noise ratio and limited sampling per cardiac cycle. Comprehensive analysis requires a sequence of operations, including ROI segmentation [18], background phase correction [19], velocity aliasing correction [20], signal extraction, and cardiac and respiratory cycles identification.

In practice, these processing steps are often performed using heterogeneous software tools and customized scripts [21, 22]. Several key operations, particularly ROI segmentation and background phase correction [7, 18, 23, 24], remain operator‐dependent and require manual adjustment or parameter tuning. Numerous automated approaches have previously been proposed for individual phase‐contrast MRI processing tasks, including threshold‐based segmentation [25], active contour methods [26], deep learning‐based frameworks [27, 28, 29], and automated background phase correction techniques [30]. However, most existing solutions target isolated processing tasks rather than offering a comprehensive post‐processing framework designed for RT‐PC MRI. Moreover, as many algorithms were originally developed for conventional CINE‐PC or cardiovascular flow MRI applications, their applicability to continuous RT‐PC MRI analysis may be limited. Consequently, RT‐PC MRI analysis often relies on multiple software packages and customized scripts, hindering workflow standardization, reproducibility, and accessibility.

Beyond workflow integration, challenges also remain in the quantification of respiratory modulation. Existing RT‐PC studies commonly quantify respiratory modulation using frequency‐domain metrics [10, 15, 17] or fixed time‐domain phase segmentation [1, 16]. However, these approaches may provide limited phase‐resolved information or assume temporal alignment between respiratory signals and flow responses. Since physiological delays between respiration and neurofluid responses may occur [31], fixed phase segmentation can underestimate modulation amplitude or incompletely characterize phase‐dependent dynamics.

These limitations highlight the need for a unified and standardized framework for RT‐PC post‐processing and phase‐resolved signal analysis.

To address these challenges, we developed Flow 2.0, a standardized toolbox for RT‐PC analysis. Flow 2.0 integrates image processing, signal extraction, time‐ and frequency‐domain analysis, and phase‐resolved respiratory modulation within a unified environment. The toolbox combines automated processing workflows with optional interactive validation and supports modular application across different flow imaging targets. Preliminary components of the toolbox have been previously presented in conference abstracts [32, 33]. In this work, we present the architecture, core processing modules, and representative applications of Flow 2.0 using RT‐PC datasets acquired under free and deep breathing conditions.

## 2 Methods

This study was approved by the local Ethics Committee (CPP Nord Ouest II, Amiens, France; reference: PI2019_843_0056) and conducted in accordance with the Declaration of Helsinki. All participants provided written informed consent.

### 2.1 Software Architecture and Workflow

Flow 2.0 was developed using Interactive Data Language (IDL) and distributed as a standalone executable application compatible with Windows and macOS platforms.

The toolbox supports 2D RT‐PC datasets stored in standard DICOM format, including DICOMDIR‐organized datasets. Compatibility with datasets from multiple major MRI vendors (e.g., Philips, Siemens, and GE) was considered during development. Physiological reference signals, including respiratory and cardiac recordings, can be integrated for analyses.

**FIGURE 1:**
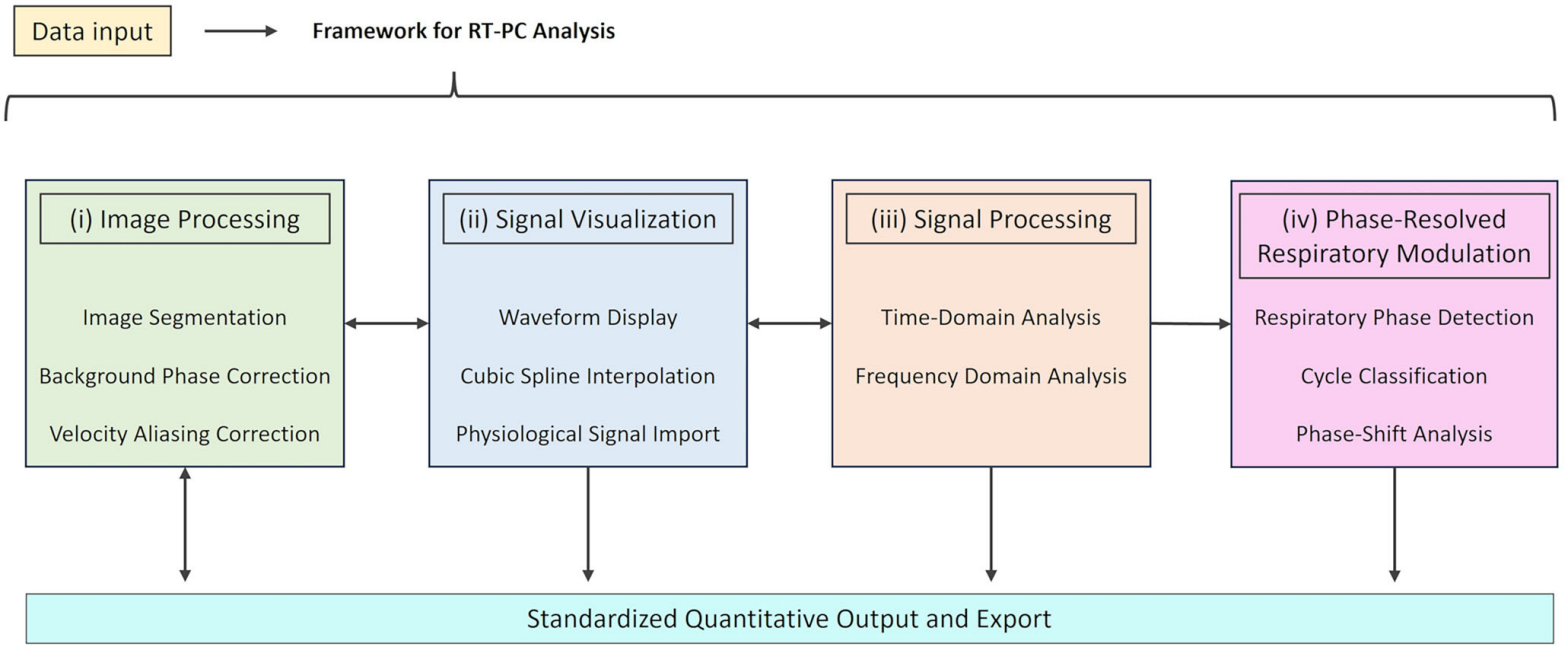
Modular architecture and processing workflow of Flow 2.0. RT‐PC data are first imported into the analysis framework, followed by target selection. The framework is structured into four core components: (i) image processing, (ii) signal visualization, (iii) signal processing, and (iv) phase‐resolved respiratory modulation. The image processing component includes image segmentation, background phase correction, and velocity aliasing correction. The signal visualization component enables waveform display and comparison, with optional integration of physiological signals. The signal processing component performs time‐domain and frequency‐domain analyses. The respiratory modulation component enables phase‐resolved analysis of RT‐PC signals relative to physiological recordings or self‐gating‐derived respiratory information, including respiratory phase detection, cycle classification, and phase‐shift analysis. Quantitative results are generated in a standardized output format for export.

The overall architecture is illustrated in Figure 1. Following data import, processing is performed within a unified interface organized into four modules: (i) image processing, (ii) signal visualization, (iii) signal processing, and (iv) respiratory modulation quantification (Figures S1 and S4). The design philosophy of Flow 2.0 emphasizes standardized and automated processing while preserving optional interactive validation.

In addition, a dedicated respiratory modulation quantification module enables phase‐resolved assessment of RT‐PC flow signals. Respiratory phase segmentation can be performed using synchronized physiological recordings (e.g., respiratory belt signals) or directly from RT‐PC signals through self‐gating approaches.

Most processing parameters can be adjusted directly through the graphical user interface without modification or recompilation of the executable.

### 2.2 Data Input

After selection of a DICOMDIR file, Flow 2.0 performs optimized parsing of DICOM metadata to identify acquisition series and extract key parameters, including matrix size, field of view, TE, TR, and VENC. Selective metadata extraction enables efficient handling of large RT‐PC datasets comprising thousands of frames.

After sequence selection, image frames and associated metadata are automatically loaded into the processing environment. A user‐defined field of view (FOV) can then be applied to restrict analysis to the target region, reducing computational load and facilitating ROI‐based processing.

### 2.3 Image Processing

Image processing in Flow 2.0 includes segmentation, background phase correction, and velocity aliasing correction. These image‐processing operations are integrated with the signal visualization and signal processing modules, enabling both frame‐wise and temporal analyses within a unified workflow.

#### 2.3.1 Automated Segmentation

Flow 2.0 provides three segmentation strategies adapted to different anatomical and motion characteristics of the target structure.

1. Cardiac Frequency–Based Segmentation

A cardiac frequency–based segmentation strategy identifies flow‐related pixels according to their temporal oscillatory characteristics rather than spatial intensity contrast [34].

For each pixel *i*, the temporal velocity signal p(t) is transformed into the frequency domain using Fourier analysis to obtain the amplitude spectrum $P_{i}\left(f\right)$. A normalized spectral energy index is then computed within the dominant cardiac‐frequency band (typically $f_{\text{dominant}}$ ± 0.2 Hz) relative to the total spectral energy within the 0.8–2.0 Hz range:

(1) $$I_{i}=\frac{\sum_{f\in \left[f_{\text{dominant}}-∆f,f_{\text{dominant}}+∆f\right]}P_{i}\left(f\right)}{\sum_{f\in \left[0.8,2\right]}P_{i}\left(f\right)}$$

where $f_{\text{dominant}}$ denotes the dominant spectral frequency within the 0.8–2.0 Hz range and ∆f was set to 0.2 Hz.

The normalized index $I_{i}$ is linearly rescaled to an eight‐bit intensity map. Higher values of $I_{i}$ reflect a stronger contribution of cardiac‐frequency components in the pixel‐wise signal. The derived frequency‐domain intensity map is subsequently segmented using a seed‐based region‐growing approach. A user‐selected seed point and an adjustable intensity threshold (default: 100/255) are used to generate the binary mask.

**FIGURE 2:**
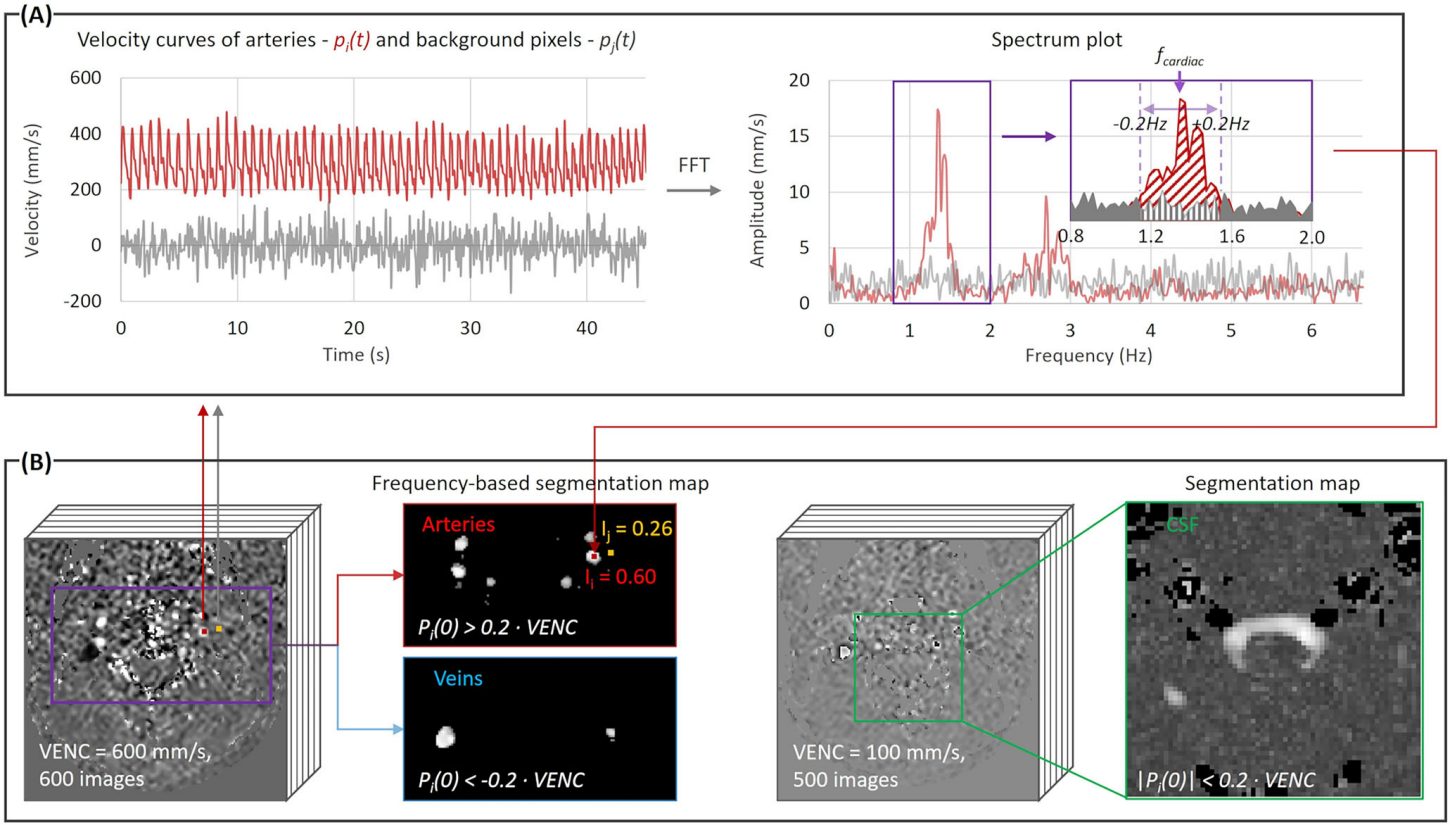
Cardiac frequency–based segmentation framework. (A) Temporal velocity signals *p(t)* from representative pixels (arterial and background) and their frequency‐domain representations. The dominant spectral frequency $f_{\text{cardiac}}$ is identified within a predefined physiological frequency range (0.8–2.0 Hz). A normalized spectral ratio $I_{i}$ is computed by integrating spectral energy within a local window (±0.2 Hz) centered on $f_{\text{cardiac}}$ and normalizing by the total energy within the predefined range. (B) The example pixels shown in (A) are mapped to their spatial locations in RT‐PC data. Pixel‐wise $I_{i}$ values are used to generate a frequency‐based segmentation map. Flow‐related regions are further classified into arteries, veins, and CSF based on the zero‐frequency component $P_{i}\left(0\right)$ relative to VENC.

Pixels are further classified according to the sign and magnitude of the zero‐frequency component $P_{i}\left(0\right)$, enabling differentiation between arterial, venous, and CSF regions (Figure 2).

2 Translation‐based ROI propagation

For structures exhibiting limited deformation but mild positional displacement, such as pulsatile vascular structures (e.g., internal carotid arteries at the C2–C3 level), a translation‐based ROI propagation strategy is applied. The ROI is propagated from the previous frame and iteratively translated within a local neighborhood to maximize instantaneous flow magnitude while preserving ROI shape and pixel count, enabling frame‐wise motion compensation.

3 Active contour segmentation

For vessels exhibiting substantial deformation or non‐rigid motion (e.g., the aorta, portal veins), an active contour model based on the classical snake formulation is applied [35]. The contour is propagated from the previous frame and used as initialization for subsequent frames, ensuring temporal continuity. Segmentation is governed by four user‐adjustable parameters controlling contour elasticity, curvature regularization, gradient attraction, and the number of convergence iterations. The default settings are Elasticity = 10, Curvature = 10, Gradient = 1, and Iterations = 30, although all parameters can be modified through the graphical user interface.

Optional frame‐wise refinement can be applied in challenging cases while preserving the overall automated workflow.

#### 2.3.2 Automated Background Phase Correction

Background phase offsets in RT‐PC data may arise from eddy currents, gradient nonlinearities, and local susceptibility effects, producing baseline velocity bias even in static tissues. To reduce these offsets, Flow 2.0 applies an automated static tissue–based correction strategy [34].

For each pixel *i*, the measured velocity signal $v_{i}\;\left(t\right)$ can be expressed as the sum of the true velocity ${v_{i}}^{\text{true}}\;\left(t\right)$ and a background offset $v^{\text{offset}}\left(t\right)$:

(2) $$\begin{array}{c} v_{i}\;\left(t\right)={v_{i}}^{\text{true}}\;\left(t\right)+v^{\text{offset}}\left(t\right) \end{array}$$

**FIGURE 3:**
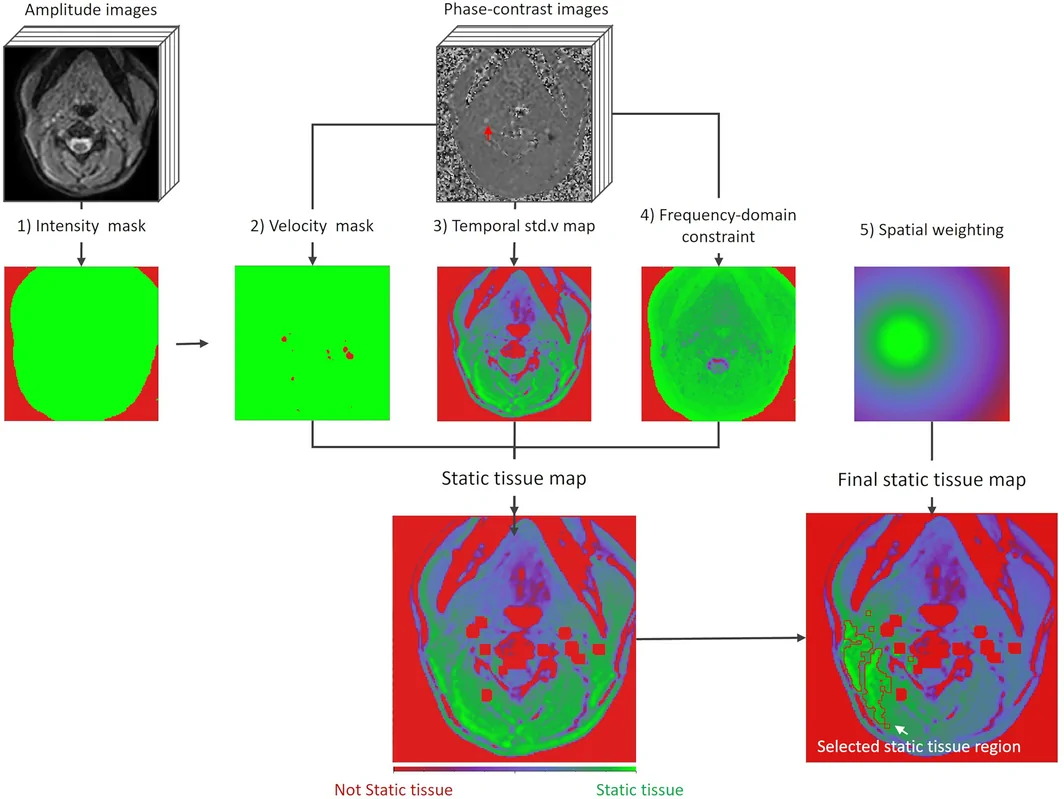
Automated background phase correction based on multi‐criteria static tissue identification. An initial amplitude‐based mask is applied to remove air regions, with excluded areas shown in red (1). A velocity‐based constraint is then applied to suppress high‐flow vascular regions (2). Temporal standard deviation (std.v) of the velocity signal is subsequently computed, where high‐variance regions (red) correspond to non‐static tissues such as bone and cavities (3). A frequency‐domain constraint is further introduced to suppress pixels exhibiting periodic components within the cardiac frequency range, including low‐flow vessels and CSF (4). The results from steps (2–4) are combined and mapped to an 8‐bit intensity image to generate an initial static tissue map. Finally, spatial weighting is applied to favor regions proximal to the target ROI (5), and high‐intensity regions (green) are selected as the final static reference region for background phase correction.

Static tissue regions are identified through a multi‐stage filtering process (Figure 3).

1. Amplitude‐based mask (air removal)

Low‐signal regions (e.g., air) are excluded using a threshold applied to the temporal mean amplitude image:

(3) $$\begin{array}{c} M_{\mathrm{amp}}\left(i\right)=\left\{\begin{array}{ll} 1, & \;{\bar{S}}_{i}^{\mathrm{amp}}>\tau_{\mathrm{amp}}\\ 0, & \;\text{otherwise} \end{array}\right. \end{array}$$

where ${\bar{S}}_{i}^{\mathrm{amp}}$ denotes the temporal mean amplitude signal of pixel *i*, and $\tau_{\mathrm{amp}}$ was empirically set to 25% of the maximum pixel intensity within the image.

2 Velocity‐based mask (flow suppression)

High‐flow regions are excluded based on the absolute value of the mean velocity:

(4) $$\begin{array}{c} \left|{\bar{v}}_{i}\right|<\tau_{v}\cdot \text{VENC} \end{array}$$

where ${\overset{`}{v}}_{i}$ is the temporal mean velocity and $\tau_{v}$ was empirically set to 0.1.

3 Temporal standard deviation map (dynamic suppression)

The temporal standard deviation of the velocity signal is computed for each pixel:

(5) $$\begin{array}{c} \sigma_{i}=\mathrm{std}\left(v_{i}\left(t\right)\right) \end{array}$$

Low‐variance regions (green) correspond predominantly to static tissues, whereas high‐variance regions (red) correspond to dynamic or non‐static structures such as bone interfaces or cavities.

4 Frequency‐domain constraint (periodic component suppression)

A frequency‐domain constraint was additionally applied to suppress residual low‐flow vessels and CSF pulsatility using the normalized spectral energy within the dominant frequency band. Pixels exhibiting strong periodic components (e.g., blood flow or CSF pulsatility) are suppressed via inverse mapping.

The resulting masks were combined to generate an initial static tissue map.

5 Spatial weighting (local prioritization)

To account for spatial variability of background phase offsets, a distance‐based spatial weighting function is applied to prioritize regions near the ROI, modeled as a smooth decay with increasing distance:

(6) $$\begin{array}{c} w\left(x,y\right)=\exp\left(-\frac{{\left(x-x_{0}\right)}^{2}+{\left(y-y_{0}\right)}^{2}}{\sigma_{d}^{2}}\right) \end{array}$$

where (*x*_*0*_, *y*_*0*_) denotes the ROI center and $\sigma_{d}$ controls the spatial decay. Local spatial weighting was used to partially account for spatial variability of background phase offsets near the target ROI.

6 Background estimation and correction

The background offset was estimated as the spatial average over the selected static tissue region:

(7) $$\begin{array}{c} v^{\text{offset}}\left(t\right)=\frac{1}{N}\;\sum_{i\in \Omega_{\text{static}}}v_{i}\left(t\right) \end{array}$$

where $\Omega_{\text{static}}$ denotes the selected static region and *N* is the number of pixels.

The corrected velocity is then obtained as follows:

(8) $$\begin{array}{c} v_{i}^{\text{true}}\left(t\right)=v_{i}\left(t\right)-v^{\text{offset}}\left(t\right) \end{array}$$

To accommodate such cases, an optional semi‐automated correction mode allows user adjustment of key processing parameters, including amplitude and velocity thresholds, ROI‐selection criteria, and internal masking (Figure S3).

#### 2.3.3 Velocity Aliasing Correction

Velocity aliasing may occur in phase‐contrast MRI when flow velocities exceed the prescribed velocity encoding (VENC), producing discontinuities in measured velocity magnitude and polarity. Flow 2.0 applies an automated aliasing correction strategy adapted from the Flow 1.0 implementation [7].

For aliased pixels, the corrected velocity $v_{\text{correct}}$ is estimated as:

(9) $$\begin{array}{c} v_{\text{correct}}=\left(2\cdot \text{VENC}-\left|v_{\text{error}}\right|\right)\cdot \left(-\frac{v_{\text{error}}}{\left|v_{\text{error}}\right|}\right) \end{array}$$

where $v_{\text{error}}$ denotes the measured aliased velocity.

For vascular flow with a predominantly unidirectional pattern, aliasing is detected using polarity consistency within the ROI. Pixels exhibiting polarity opposite to the dominant ROI direction are identified as aliased and corrected accordingly.

**FIGURE 4:**
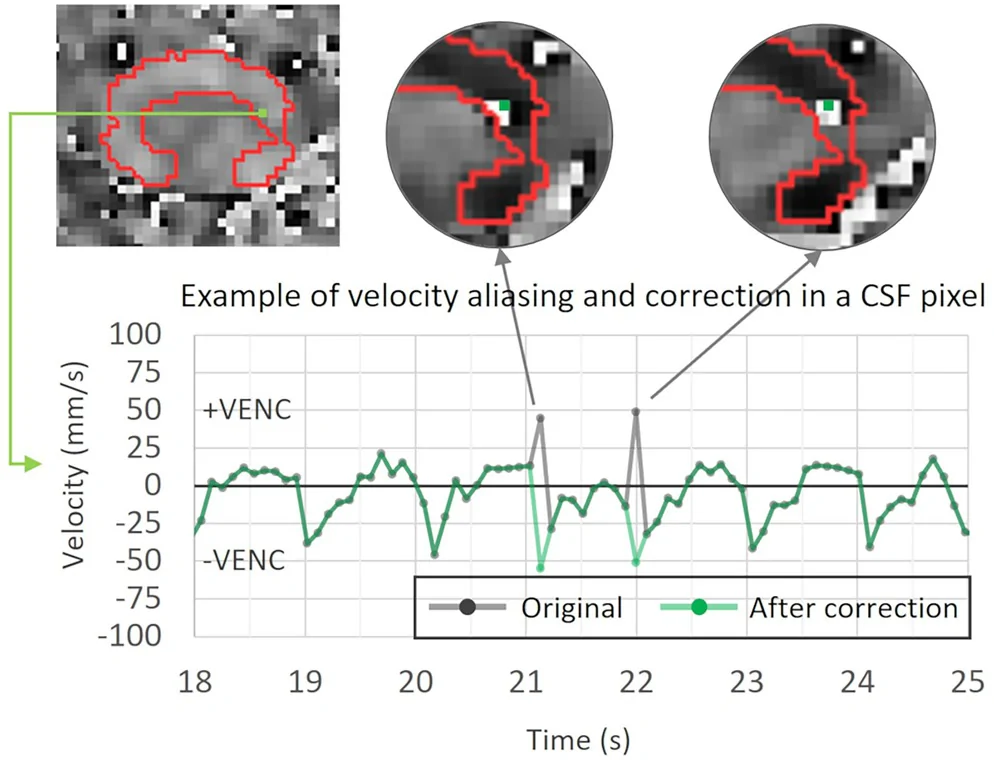
A representative CSF ROI is shown (top left), with the aliased pixel highlighted in green. The gray curve represents the measured velocity waveform of this pixel. Two aliased time frames are indicated, and the corresponding phase‐contrast images are shown in the circular insets. In these frames, flow that should appear in the negative direction is incorrectly displayed as high‐intensity positive velocity due to aliasing. The corrected waveform (green) shows the velocity signal after aliasing correction.

For oscillatory flows such as CSF, where velocity direction varies over time, a frame‐wise consistency criterion is applied. Pixel polarity is compared with the mean ROI polarity at the corresponding time frame, and inconsistent pixels are corrected as potential aliasing artifacts (Figure 4).

### 2.4 Signal Visualization

The signal visualization module supports displaying ROI‐derived time‐resolved signals and integrating external physiological data (Figure S1B). The module links image‐level processing with signal‐level analysis and supports downstream respiratory modulation and coupling analysis.

#### 2.4.1 Waveform Visualization and Comparison

For each ROI, multiple quantitative parameters can be extracted and visualized as time‐resolved signals, including flow, velocity, cross‐sectional area, and spatial position and displacement.

Selected signals can optionally undergo cubic spline interpolation to increase effective sampling density and reduce underestimation of peak amplitudes in the cardiac frequency signals. Interpolated signals are intended for visualization and waveform reconstruction rather than replacement of the original RT‐PC measurements.

Two signals can be jointly visualized within a shared temporal framework, for example to compare flow and respiratory signals (Figure S2). The secondary signal can be linearly rescaled and baseline‐shifted relative to a primary reference using automatic or manual adjustment.

Waveform displays are linked to corresponding image frames, enabling verification of ROI measurements and inspection of frame‐specific features such as aliasing. Outputs from the signal processing module, including filtered signals and segmentation points, can be displayed within the same visualization environment.

#### 2.4.2 Physiological Signal Import

External physiological signals can be imported and temporally aligned with image‐derived signals. When supported acquisition formats are available (e.g., Philips physiological trace files), synchronization is performed automatically. User‐defined time–amplitude vectors can also be imported to integrate respiratory, cardiac, or other periodic reference signals.

### 2.5 Signal Processing

**FIGURE 5:**
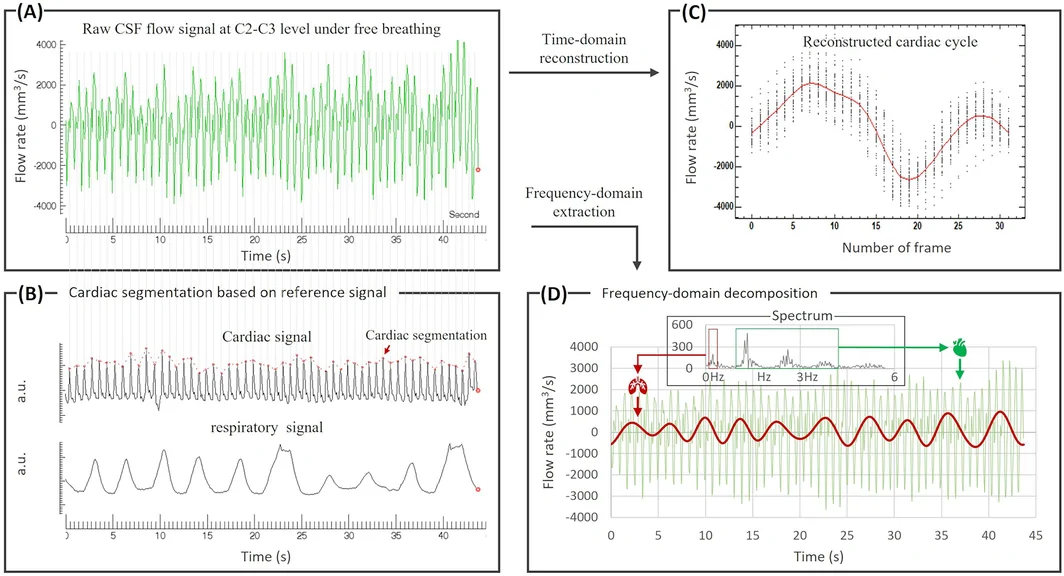
Unified time‐ and frequency‐domain signal processing framework. (A) Raw CSF flow signal acquired at the C2–C3 level during free breathing. (B) Segmentation points are automatically defined using physiological reference signals. In this example, cardiac signal extrema are used as segmentation points and applied to the flow signal. (C) Cycle‐wise reconstructed cardiac flow waveform with 32 standardized phase points and the distribution of sampled data at each phase (Reconstructed cardiac cycle). (D) Frequency‐domain analysis and extraction of specific frequency components through spectral selection and reconstruction of the corresponding frequency bands.

The signal processing module supports time‐ and frequency‐domain analysis of physiological signals, including cycle‐resolved quantification and frequency‐specific signal decomposition (Figure 5).

#### 2.5.1 Time‐Domain Segmentation and Reconstruction

Cycle segmentation is performed using reference physiological signals (Figure 5B) or directly derived from RT‐PC time‐series signals (Figure S1). Direct segmentation from RT‐PC signals enables self‐gating without external physiological recordings, whereas physiological signals allow cycle segmentation to be synchronized across separate RT‐PC acquisitions, facilitating comparison between blood flow and CSF signals. Segmentation points identified from the reference signal are subsequently applied to the target RT‐PC signal for phase‐aligned analysis.

In this study, cardiac signals were used as a representative example. Segmentation points were automatically detected using a step‐wise extremum search strategy based on signal periodicity. The dominant cycle length $N_{c}$ (in samples) was estimated from the signal periodicity, and segmentation points $t_{k}$ were recursively identified as:

(10) $$\begin{array}{c} t_{k}=\arg{\mathrm{ext}}_{t\in \left[t_{k}-N_{c}-∆,t_{k}-N_{c}+∆\right]}s\left(t\right),\;k=1,2,\ldots \end{array}$$

where s(t) denotes the reference signal, ext represents a local extremum operator (minimum or maximum, depending on signal polarity), and Δ defines the search‐window half width (typically $N_{c}/2$). This iterative strategy improves robustness to cycle‐to‐cycle variability.

Following segmentation, individual cycles are resampled using cubic spline interpolation to a fixed number of phase points (default: 32), generating a standardized waveform representation (Figure 5C). This approach facilitates comparison of RT‐PC waveforms across acquisitions and conditions, while partially mitigating apparent peak underestimation associated with limited temporal sampling.

Quantitative cycle‐derived parameters are subsequently extracted, including maximum, minimum, mean value, peak‐to‐peak amplitude, positive and negative integrals (e.g., stroke volume for flow signals), net integral, and cycle duration. Upper and lower envelopes derived from cycle‐wise extrema can also be used to visualize amplitude modulation over time.

#### 2.5.2 Frequency‐Domain Analysis and Extraction

Frequency‐domain analysis is performed using fast Fourier transform (FFT) of the full time‐series signal (Figure 5D), with optional removal of the DC component. Specific frequency bands can then be retained for extraction of specific physiological signal components.

Frequency selection is performed while preserving conjugate (Hermitian) symmetry of the spectrum prior to inverse FFT reconstruction. This strategy minimizes artificial phase shifts or waveform distortions associated with asymmetric spectral truncation.

### 2.6 Phase‐Resolved Analysis of Respiratory Modulation

**FIGURE 6:**
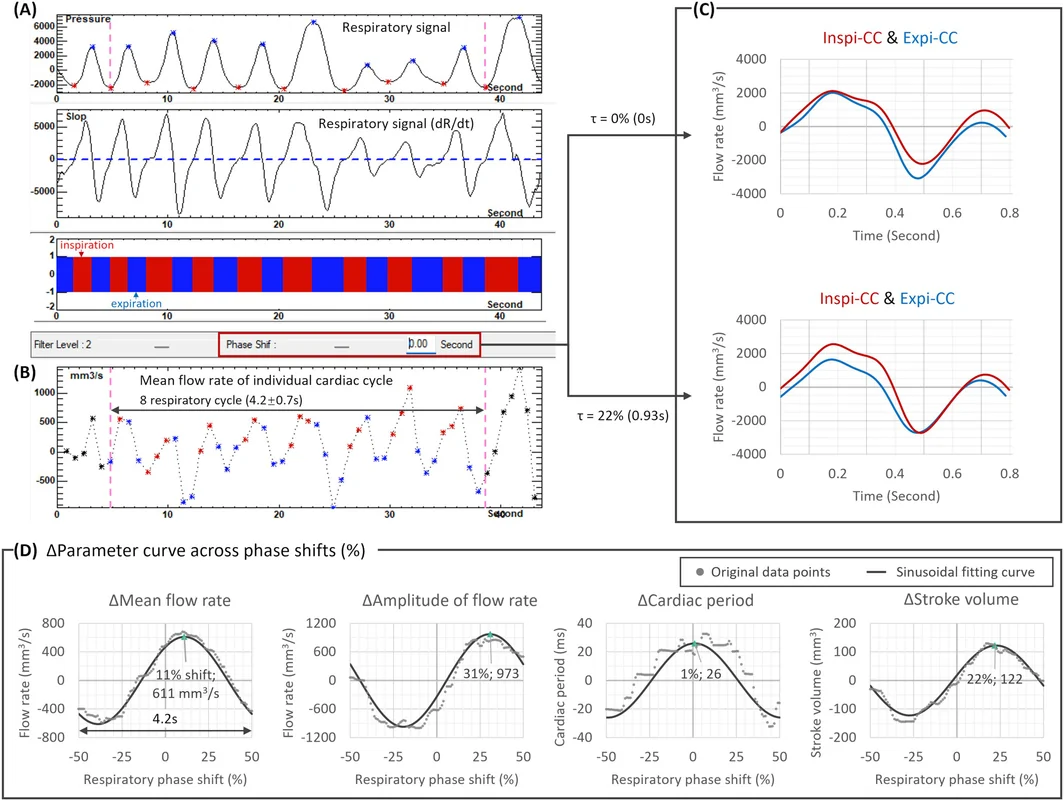
Phase‐resolved quantification of respiratory modulation in CSF flow during free breathing. (A) Automatic identification of respiratory phases based on the first derivative of the respiratory signal. Inspiration and expiration are labeled in red and blue, respectively. (B) Classification of cardiac cycles according to respiratory phase. Multiple cycle‐specific parameters can be computed for each cardiac cycle; here, cycle‐averaged flow rate is shown as an example, with red and blue markers indicating inspiration (Inspi‐CC) and expiration (Expi‐CC), respectively. (C) Reconstruction of phase‐specific cardiac cycles, showing inspiration‐averaged (Inspi‐CC) and expiration‐averaged (Expi‐CC) waveforms under different phase shifts τ. Examples with τ = 0% and τ = 22% are illustrated. (D) Phase‐resolved modulation of cycle‐specific parameters across respiratory phase shifts. For each phase shift τ, parameters are averaged over cardiac cycles within inspiration and expiration, and their difference $\Delta P\left(\tau \right)=P_{\text{insp}}\left(\tau \right)-P_{\exp}\left(\tau \right)$ is computed. Curves show ΔMean flow rate, ΔAmplitude, ΔCardiac period, and ΔStroke volume. For each parameter, a respiratory‐cycle–wide segment is extracted from the full scanning range and expressed in normalized respiratory phase (%). Sinusoidal fitting is used to estimate the maximal modulation amplitude and the corresponding phase.

The respiratory modulation module (Figures 6 and S4) supports phase‐resolved analysis of respiration‐related variations in ROI‐derived RT‐PC signals. An earlier implementation of this analytical approach has been previously described [34].

#### 2.6.1 Respiratory Phase Detection

Respiratory phase segmentation is performed using externally recorded respiratory signals (Figure 6A). The first derivative of the respiratory waveform is computed, and derivative zero‐crossings are used to define inspiration and expiration boundaries.

Optional signal smoothing and removal of closely spaced detections can be applied to improve robustness against noise and transient respiratory fluctuations. Nevertheless, highly irregular respiratory patterns may still affect the accuracy of derivative‐based phase detection.

Optional derivative thresholding can additionally identify reduced‐slope intervals corresponding to respiratory hold phases. Detected respiratory intervals can also be interactively adjusted when necessary.

#### 2.6.2 Cardiac Cycle Classification Across Respiratory Phases

Following respiratory phase identification, cardiac cycles are assigned to a respiratory phase according to their temporal position relative to the respiratory signal (Figure 6B).

Cycle‐derived parameters including mean value, peak amplitude, cycle duration, and stroke volume are subsequently computed for each cardiac cycle. For CSF oscillatory flow signals, stroke volume was estimated from the positive and negative flow components of each cardiac cycle as follows:

(11) $$\begin{array}{c} \mathrm{SV}=\frac{1}{2}\left(\int_{Q\left(t\right)>0}Q\left(t\right)+\left|\int_{Q\left(t\right)<0}Q\left(t\right)\right|\right) \end{array}$$

where Q(t) denotes the flow rate waveform. Prior to integration, the waveform was upsampled using 10× cubic spline interpolation to improve numerical stability.

Figure 6B illustrates the representative distributions of cycle‐averaged flow values across respiratory phases. Phase‐specific cardiac waveforms were reconstructed by averaging cardiac cycles within each respiratory phase (Figure 6C). This cycle‐based representation enables comparison of respiratory‐related waveform variations across respiratory conditions.

#### 2.6.3 Phase‐Shift Analysis

A phase‐shift analysis was performed to evaluate respiration‐related parameter variations across temporal offsets between respiratory signals and RT‐PC measurements (Figure 6C,D). Respiratory phase boundaries were systematically shifted in time, and cardiac cycle classification and parameter extraction were repeated for each phase shift.

Phase shifts τ were scanned over the range τϵ[−4s,4s] with a step size of 0.01 s. This range is chosen to ensure coverage of at least one full respiratory cycle under typical breathing conditions. For each phase shift τ, cycle‐derived parameters were averaged separately for inspiration‐ and expiration‐associated cardiac cycles:

(12) $$\begin{array}{c} P_{\text{inspi}}\left(\tau \right)=\frac{1}{N_{\text{inspi}}\left(\tau \right)}\sum_{\mathrm{k\epsilon}\text{Inspi}\left(\tau \right)}P_{k} \end{array}$$

(13) $$\begin{array}{c} P_{\text{expi}}\left(\tau \right)=\frac{1}{N_{\text{expi}}\left(\tau \right)}\sum_{\mathrm{k\epsilon}\text{Expi}\left(\tau \right)}P_{k} \end{array}$$

where $P_{k}$ denotes the parameter value of the k‐th cardiac cycle, and $N_{\text{insp}}$, $N_{\exp}$ denote the corresponding numbers of classified cycles.

The respiration‐induced modulation is then defined as follows:

(14) $$\begin{array}{c} \Delta P\left(\tau \right)=P_{\text{inspi}}\;\left(\tau \right)-P_{\text{expi}}\;\left(\tau \right) \end{array}$$

ΔP(τ) describes the parameter variations as a function of respiratory phase shift.

For standardized representation and comparison across parameters, one respiratory cycle ($T_{\text{resp}}$) segment was extracted from the full phase‐shift range and expressed in normalized respiratory phase (%), as shown in Figure 6D, whereas modulation amplitudes are reported in their original physical units by default. When required, normalized modulation metrics can be readily derived during subsequent analysis using the corresponding mean parameter values provided by the software outputs, thereby facilitating comparisons across subjects and parameters.

Optionally, ΔP(τ) can be approximated using a sinusoidal model to estimate the modulation amplitude and corresponding phase. The coefficient of determination (*R* ^2^) is additionally computed to quantify the goodness‐of‐fit between the measured modulation curve and the sinusoidal approximation. This fitting is intended as a simplified descriptive representation of respiratory modulation patterns while providing a compact quantification of the respiratory effect.

### 2.7 Standardized Quantitative Output and Export

To support reproducibility and downstream analysis, quantitative outputs are systematically standardized and exported. Outputs include time‐series waveforms, cycle‐derived parameters, and phase‐resolved reconstructions generated by the processing framework.

Outputs are organized using consistent naming conventions and physical units to facilitate comparison across acquisitions, subjects, and processing conditions.

Intermediate processing elements, including ROI definitions and background phase correction masks, can also be preserved and reloaded to support consistent reapplication of identical processing workflows.

User‐defined confidence levels (Trust Level, 1–5) can optionally be assigned to processed datasets and preserved during data export to facilitate quality tracking and downstream analysis. Exported data are provided in spreadsheet‐compatible formats to support external statistical analysis and interoperability with other processing environments.

### 2.8 Software Availability

Flow 2.0 is freely available for academic use via the official project website (https://bioflowdynamics.com/software‐flow2), which provides documentation, tutorial materials, and anonymized example datasets used in this study. Example datasets include RT‐PC acquisitions of cerebral blood flow and CSF under free‐ and deep‐breathing conditions. The software will continue to be maintained and updated by the authors through the BioFlow Dynamics platform following publication.

## 3 Example Application

**FIGURE 7:**
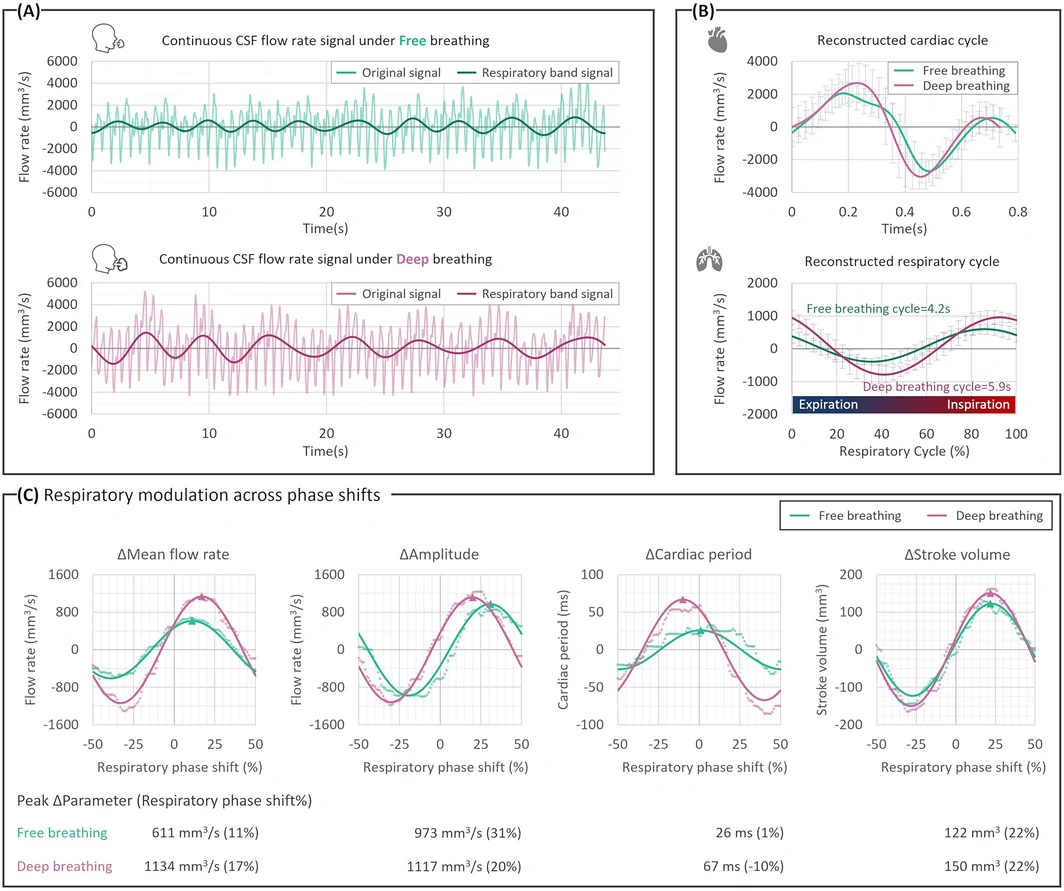
Respiratory modulation of CSF flow under free and deep breathing. (A) Representative continuous CSF flow signals during free breathing (top) and deep breathing (bottom). Light traces show the original signal, and darker curves indicate the extracted respiratory‐band component. (B) Reconstructed cardiac and respiratory cycles for the two breathing conditions. The upper panel shows the mean reconstructed cardiac cycles for free breathing (green) and deep breathing (purple), with error bars representing the standard deviation across independent cardiac cycles. The lower panel shows the reconstructed mean respiratory cycles derived from the respiratory‐band signal, with average cycle durations of approximately 4.2 s (free breathing) and 5.9 s (deep breathing). (C) Phase‐shift–dependent inspiration–expiration differences (ΔParameter) across the respiratory cycle for mean flow rate, flow amplitude, cardiac period, and stroke volume. Dots represent measured values at each tested phase shift, and solid curves indicate the sinusoidal fit. The table summarizes the maximal modulation amplitude and the corresponding respiratory phase shift under both breathing conditions.

The Flow 2.0 toolbox was applied to the representative CSF RT‐PC dataset provided with the software, acquired in a healthy young male volunteer at the C2–C3 level under free and sustained deep breathing conditions (Figure 7). A complete demonstration of the processing workflow under sustained deep breathing is provided in Video S1.

### 3.1 Respiratory Modulation of CSF Flow

Representative CSF flow signals and respiratory‐band components are shown in Figure 7A for both breathing conditions. Increased respiratory oscillation amplitudes were observed during sustained deep breathing compared with free breathing.

Reconstructed cardiac‐cycle and respiratory‐cycle waveforms are shown in Figure 7B. Differences in respiratory and cardiac cycle durations were observed between conditions, while the overall waveform morphology remained comparable.

The phase‐resolved analysis module enabled visualization of respiratory modulation across multiple parameters, including mean flow rate, amplitude, cardiac period, and stroke volume (Figure 7C). The resulting ΔParameter curves showed phase‐dependent variations across respiratory phase shift.

Phase shifts between respiratory signals and flow‐derived parameters could also be visualized using the phase‐resolved analysis module.

### 3.2 Application to Previously Validated RT‐PC Analyses

Flow 2.0 processing strategies have been applied in multiple RT‐PC studies, including validation against conventional CINE‐PC measurements and phantom experiments [36], as well as investigations of arterial, venous, and CSF flow dynamics [31, 37, 38], CSF–cerebral blood flow coupling analysis [2, 10, 39], and the development of new physiology‐related quantification metrics [40, 41].

## 4 Discussion

This study presents Flow 2.0, a standardized toolbox for RT‐PC designed to support reproducible and multi‐parametric analysis of neurofluid dynamics across time and frequency domains. The toolbox integrates image processing, signal extraction, and phase‐resolved analysis within a unified processing environment.

### 4.1 Integrated RT‐PC Framework for Standardized Post‐Processing

Although RT‐PC MRI enables characterization of multi‐frequency neurofluid dynamics, broader adoption remains limited by fragmented and labor‐intensive post‐processing workflows [42, 43, 44].

Flow 2.0 addresses this challenge by integrating image processing, waveform visualization, and signal‐domain analysis within a standardized environment, enabling more consistent parameter extraction and reproducible analysis across datasets and experimental conditions.

### 4.2 Automation With Interactive Oversight to Reduce Operator Variability

Operator‐dependent variability remains a major challenge in RT‐PC post‐processing, particularly for long time‐series acquisitions. Flow 2.0 reduces this variability through automated implementation of segmentation, background phase correction, aliasing correction, and cycle detection [45, 46], while preserving optional interactive validation and user oversight. Optional interactive validation enables users to refine ROI boundaries or review cycle detection when necessary, supporting analytical reliability while maintaining standardized processing workflows.

### 4.3 Phase‐Resolved Quantification of Respiratory Modulation

Respiratory modulation of RT‐PC signals is commonly evaluated using fixed phase segmentation or frequency‐domain averaging [1, 16]. Flow 2.0 extends these by enabling phase‐resolved analysis across respiratory phase shifts and multiple flow‐derived parameters.

The phase‐resolved analysis module allows visualization of parameter variations across respiratory phase offsets and supports comparative analysis of multiple signal‐derived metrics (Figure 7C).

Similar phase‐resolved concepts have been explored in previous RT‐PC and respiratory‐resolved flow imaging studies [31, 37, 38, 47].

### 4.4 Modular and Transferable Architecture Beyond Neurofluid Applications

Many RT‐PC post‐processing workflows remain application‐specific, limiting portability across vascular territories and imaging targets [1, 42, 48].

In contrast, Flow 2.0 is designed as a modular and anatomically agnostic framework. Its core processing modules operate on generic RT‐PC velocity data and can therefore be applied to a wide range of flow imaging applications. Preliminary use in portal venous flow analysis further illustrates this adaptability [49].

By decoupling processing logic from anatomical context, the framework enables broader application of standardized RT‐PC analysis beyond neurofluid imaging.

### 4.5 Limitations and Future Work

Despite its integrated design, several limitations remain.

The current aliasing correction algorithm primarily addresses single‐wrap conditions and may remain limited in regions affected by severe partial‐volume effects or complex multi‐wrap phase behavior, where more advanced phase‐unwrapping approaches may be required [50]. Extension to multi‐wrap phase unwrapping will be an important direction for future development. In addition, the polarity‐based correction strategy assumes predominantly unidirectional flow within the ROI and may be less reliable in the presence of complex flow patterns, local velocity reversals, or severe noise contamination.

In addition, active contour segmentation parameters currently remain predefined and may require further optimization across different flow environments.

Future development will focus on expanding the range of available processing strategies within the framework. Potential additions include low‐order polynomial and spline‐based background phase correction methods [24], adaptive parameter optimization, and AI‐assisted segmentation approaches. Such developments may further improve flexibility and facilitate application across a broader range of RT‐PC datasets and anatomical regions.

Although the framework supports datasets from multiple vendors, differences in acquisition formats and metadata structures persist. Further cross‐platform validation will therefore be necessary to support broader adoption.

Finally, while initial applications beyond neurofluid imaging have been explored, systematic validation in other vascular territories remains to be established.

## 5 Conclusion

Flow 2.0 provides a standardized toolbox for real‐time phase‐contrast MRI, enabling reproducible and efficient analysis of neurofluid dynamics across time and frequency domains. By integrating segmentation, background phase correction, velocity aliasing correction, and signal‐domain analysis within a unified environment, the toolbox addresses key limitations of fragmented RT‐PC workflows.

The phase‐resolved analysis module supports multi‐parametric evaluation of respiratory‐related signal variations and their phase‐dependent temporal characteristics.

Although initially developed for neurofluid applications, the modular design of Flow 2.0 supports broader use across RT‐PC flow imaging applications.

## Funding

This research was funded by the France National Research Agency (reference: Hanuman ANR‐18‐CE45‐0014, CALCOCIMO ANR‐23‐CE18‐0026 and EquipEX FIGURES 10‐EQUATION PX‐0001).

## Conflicts of Interest

The authors declare that the research was conducted in the absence of any commercial or financial relationships that could be construed as a potential conflicts of interest.

## Supporting information


**Figure S1:** Main analysis interface of Flow 2.0 for RT‐PC post processing. (A) Image processing module. The internal carotid artery is shown as an example, where automated frequency‐based segmentation is used to define the region of interest (ROI) for subsequent quantitative analysis. (B) Signal visualization module. Time‐series signals derived from the selected ROI are displayed, illustrated here by the flow rate waveform. Cardiac segmentation points are overlaid (red markers). Interactive cursor control enables synchronized navigation between waveform positions and corresponding image frames, allowing direct verification of segmentation and frame‐specific flow parameters with real‐time display of corresponding measurements. (C) Time‐domain analysis component of the signal processing module. Cardiac cycle segmentation, cycle‐specific parameter extraction, and reconstruction of the mean cardiac cycle waveform are performed. The white plot represents the mean flow value of each individual cardiac cycle across the time series, while the reconstructed average cardiac cycle waveform is shown in red in the right panel.
**Figure S2:** Example of signal visualization. Respiratory modulation module. An example is shown for flow extracted from a right internal jugular vein ROI (black curve) together with a respiratory signal (red curve). The eye icon denotes the signal currently displayed (flow rate). The upper panels illustrate the ROI location on the phase‐contrast image and its magnified view. The visualization module enables time‐resolved representation of multiple ROI‐derived parameters and supports joint display of different signals within a shared temporal framework. Linear rescaling and baseline shifting of the secondary signal allow amplitude alignment between signals, facilitating observation of respiration‐driven flow variations. In addition, the module provides linkage between signal representation and image frames, as well as integration of multi‐source signals, supporting a unified signal analysis environment.
**Figure S3:** Semi‐automated background phase correction interface. The semi‐automated background phase correction module allows interactive refinement of static‐tissue selection used for background offset estimation. ROI Size controls the final size of the selected static‐tissue region. Inner Ring defines an optional internal mask to exclude central structures (e.g., the spinal canal) from static‐tissue selection. Outer Ring defines the search region for static‐tissue selection around the target ROI, favoring nearby tissues for background offset estimation. Mean Velocity specifies the velocity threshold used to suppress flow‐related pixels, expressed as a percentage of VENC. Amplitude Intensity Min and Max define the intensity range applied to the amplitude image for static‐tissue identification. The color map indicates the estimated reliability of candidate static‐tissue regions, ranging from poor (red) to good (green).
**Figure S4:** Respiratory modulation module. The reconstructed cardiac‐cycle waveforms were derived from the right internal carotid artery using physiological cardiac signal–based cycle segmentation. (A) Automatic respiratory phase detection interface. The upper panels display the raw respiratory waveform and its first derivative. Inspiration and expiration intervals are automatically identified based on derivative extrema and visualized using color‐coded phase bars. (B) Cardiac cycle‐wise mean flow classified by respiratory phase (red: inspiration; blue: expiration). Dashed lines indicate the selected complete respiratory cycles (cycles 1–6). The information panel displays the number of cycles and the mean respiratory period. (B1) Other cardiac cycle–derived parameters (e.g., amplitude, cardiac period, pulsatility index) can be classified and displayed in the same manner. (C) Reconstructed inspiration‐averaged (Inspi‐CC), expiration‐averaged (Expi‐CC), and total cardiac cycles using a standardized sampling scheme. (D) Mean flow of inspiration (red) and expiration (blue) cardiac cycles as a function of respiratory phase shift, obtained by systematic reclassification across temporal offsets. (E) Inspiration–expiration mean flow difference as a function of respiratory phase shift. Solid line: measured values; dashed line: sinusoidal fit. The horizontal axis is automatically constrained to ±1 mean respiratory cycle. The maximal modulation amplitude, corresponding optimal phase shift, and fitting coefficient of determination (R^2^) are reported.

**Video S1:** Demonstration of the Flow 2.0 post‐processing workflow. This video illustrates the complete post‐processing workflow using a representative C2–C3 CSF RT‐PC dataset acquired under sustained deep breathing, including data import, image processing, signal visualization, signal processing, respiratory modulation quantification, and data export.

## Data Availability

The Flow 2.0 software described in this study is freely available for academic use through the official project website (https://bioflowdynamics.com/software‐flow2/), which also provides documentation, tutorial materials, and demonstration datasets.
