# Supplementary material for: Flow 2.0: A Standardized Toolbox for Real‐Time Phase‐Contrast MRI of Neurofluid Dynamics
Source: Magn Reson Med. 2026 Jul 22;96(5):2092–105. doi: 10.1002/mrm.70519 (PMC13527278; doi:10.1002/mrm.70519)
Supplement: Supplementary file 1 [file MRM-96-2092-s001.docx]

Supplementary

## S1. Main analysis interface of Flow 2.0 for RT-PC post processing.

Figure S1. Main analysis interface of Flow 2.0 for RT-PC post processing. (A) Image processing module. The internal carotid artery is shown as an example, where automated frequency-based segmentation is used to define the region of interest (ROI) for subsequent quantitative analysis. (B) Signal visualization module. Time-series signals derived from the selected ROI are displayed, illustrated here by the flow rate waveform. Cardiac segmentation points are overlaid (red markers). Interactive cursor control enables synchronized navigation between waveform positions and corresponding image frames, allowing direct verification of segmentation and frame-specific flow parameters with real-time display of corresponding measurements. (C) Time-domain analysis component of the signal processing module. Cardiac cycle segmentation, cycle-specific parameter extraction, and reconstruction of the mean cardiac cycle waveform are performed. The white plot represents the mean flow value of each individual cardiac cycle across the time series, while the reconstructed average cardiac cycle waveform is shown in red in the right panel.

## S2. Example of the signal visualization module.

Figure S2. Example of signal visualization. Respiratory modulation module. An example is shown for flow extracted from a right internal jugular vein ROI (black curve) together with a respiratory signal (red curve). The eye icon denotes the signal currently displayed (flow rate). The upper panels illustrate the ROI location on the phase-contrast image and its magnified view. The visualization module enables time-resolved representation of multiple ROI-derived parameters and supports joint display of different signals within a shared temporal framework. Linear rescaling and baseline shifting of the secondary signal allow amplitude alignment between signals, facilitating observation of respiration-driven flow variations. In addition, the module provides linkage between signal representation and image frames, as well as integration of multi-source signals, supporting a unified signal analysis environment.

## S3. Semi-automated background phase correction interface.

Figure S3. Semi-automated background phase correction interface. The semi-automated background phase correction module allows interactive refinement of static-tissue selection used for background offset estimation. ROI Size controls the final size of the selected static-tissue region. Inner Ring defines an optional internal mask to exclude central structures (e.g., the spinal canal) from static-tissue selection. Outer Ring defines the search region for static-tissue selection around the target ROI, favoring nearby tissues for background offset estimation. Mean Velocity specifies the velocity threshold used to suppress flow-related pixels, expressed as a percentage of VENC. Amplitude Intensity Min and Max define the intensity range applied to the amplitude image for static-tissue identification. The color map indicates the estimated reliability of candidate static-tissue regions, ranging from poor (red) to good (green).

## S4. Respiratory modulation module of Flow 2.0.

Figure S4. Respiratory modulation module. The reconstructed cardiac-cycle waveforms were derived from the right internal carotid artery using physiological cardiac signal–based cycle segmentation. (A) Automatic respiratory phase detection interface. The upper panels display the raw respiratory waveform and its first derivative. Inspiration and expiration intervals are automatically identified based on derivative extrema and visualized using color-coded phase bars. (B) Cardiac cycle–wise mean flow classified by respiratory phase (red: inspiration; blue: expiration). Dashed lines indicate the selected complete respiratory cycles (cycles 1–6). The information panel displays the number of cycles and the mean respiratory period. (B1) Other cardiac cycle–derived parameters (e.g., amplitude, cardiac period, pulsatility index) can be classified and displayed in the same manner. (C) Reconstructed inspiration-averaged (Inspi-CC), expiration-averaged (Expi-CC), and total cardiac cycles using a standardized sampling scheme. (D) Mean flow of inspiration (red) and expiration (blue) cardiac cycles as a function of respiratory phase shift, obtained by systematic reclassification across temporal offsets. (E) Inspiration–expiration mean flow difference as a function of respiratory phase shift. Solid line: measured values; dashed line: sinusoidal fit. The horizontal axis is automatically constrained to ±1 mean respiratory cycle. The maximal modulation amplitude, corresponding optimal phase shift, and fitting coefficient of determination (R²) are reported.

The reconstructed total cardiac-cycle waveform shown in panel C differs from the waveform reconstructed in Figure S1C primarily because different cycle-segmentation strategies were used. Figure S1C used self-gating segmentation directly derived from the RT-PC waveform, resulting in cycles defined from peak to peak, whereas Figure S4 used external physiological cardiac signals for cycle segmentation, resulting in waveform peaks centered within the reconstructed cardiac cycle. Despite these visual differences, the reconstructed amplitudes remained similar between the two examples (approximately 2.8 mL/s in Figure S1C and 2.7 mL/s in Figure S4C).
